# A Low Sampling Rate Receiver Design for Multi-Antenna Multi-User OFDM Systems

**DOI:** 10.3390/e24040448

**Published:** 2022-03-24

**Authors:** Zeliang Ou, Xiaofeng Liu, Hongwen Yang

**Affiliations:** 1 School of Wireless Communication Center, Beijing University of Posts and Telecommunications, Beijing 100876, China; ouzeliang@bupt.edu.cn (Z.O.); yanghong@bupt.edu.cn (H.Y.); 2 China Academy of Information and Communications Technology, Beijing 100191, China

**Keywords:** low sampling rate, zero-forcing precoding, OFDM system

## Abstract

The future 6G mobile communication network will support an unprecedented amount of Internet of Things (IoT) devices, which will boost the demand for low cost terminals under the principle of green communication. One of the critical issues for low cost terminals is the sampling rate of analog-to-digital converters (ADCs) at the receivers. A high sampling rate of the ADC gives rise to a high energy consumption and high hardware cost for the terminal. In the conventional multi-user OFDM systems, all users have to sample the received signal with a sampling rate that is larger than or equal to the Nyquist rate, despite only a small fraction of the bandwidth (number of subcarriers) is allocated to each user. This paper proposes a low sampling rate receiver design for multi-antenna multi-user OFDM systems. With the aid of zero-forcing precoding, the sampling rate of the receiver can be reduced to 1/K of the Nyquist rate, where *K* is the number of users. The simulation results show that with a significant reduction in sampling rate, performance loss is insignificant and acceptable in terms of bit error rate, mutual information and peak-to-average power ratio.

## 1. Introduction

The sixth generation (6G) mobile communication network is envisioned to support the seamless connection of Internet of Things (IoT) devices [1], the scale of which will be far larger than ever before. This large-scale connection of devices will consist of smart sensors of all kinds that are closely related to the whole society; for example, advanced driving automation systems, forest fire detection and healthcare IoT [1,2]. With the boost in data produced by the IoT devices at high rates, processing them in an efficient and energy saving way could turn out to be a severe challenge [3].

A multitude of state-of-the-art technologies have been proposed to fulfill the above requirements. Orthogonal frequency-division multiplexing (OFDM) is a modulation scheme for wideband systems, which is still regarded as a key physical layer technology in 6G communication systems [4,5,6]. OFDM can realize the parallel transmission of high speed data by frequency-division multiplexing and resist multi-path attenuation. However, the disadvantages of OFDM should not be neglected. A high sampling rate and a high resolution are required on both the transmitting side and the receiving side due to the wide bandwidth of OFDM systems [7]. Furthermore, analog-to-digital converters (ADCs) at high sampling rates generate a large amount of data that needs to be processed in real time. The expensive ADCs are perceived as a heavy load for the future communication network that will use multiple antennas [8,9].

The so-called *low cost* ADCs mainly focus on two factors: low resolution quantization and low sampling rates [10]. These inevitably cause a certain amount of deterioration in the performance. However, low cost ADCs have still drawn attention from researchers around the world in recent years [11]. To further improve the performance of the whole system, besides employing purely low bit quantization [12], some researchers have investigated a mixed ADC architecture, in which some of the antennas were equipped with low resolution ADCs [13,14,15]. It should be noted that low complexity receivers with low resolution ADCs have been developed very quickly in recent years through a variety of studies [16,17,18,19,20] utilizing message passing algorithms to design near-optimal low complexity receivers, which have made it possible to implement low resolution ADCs.

This paper concentrates on a low sampling rate receiver design for OFDM systems. For traditional OFDM systems, the receiver is usually equipped with an ADC working at a sampling rate that is larger than or equal to the Nyquist rate $f_{0}=N/T$, where *N* is the number of subcarriers and 1/T is the subcarrier spacing. To reduce the sampling rate, Ref. [21] investigated a delay-division multiplexing (DDM) scheme for orthogonal frequency-division multiple access (OFDMA) passive optical networks (OPNs), which deployed an ADC operating at a sampling rate below the Nyquist rate for each optical network unit. The sampling instant of the ADC was critical in the selection of the required data and thus, a method was proposed to carry out the adjustments required for the sampling delay to demodulate the expected data. Ref. [22] proposed a simple DDM scheme for OFDMA-OPNs and 2X2 wireless multi-input multi-output (MIMO), which enabled both wired and wireless users to receive the target data with a sub-Nyquist sampling rate ADC without the demodulation of all signals. Ref. [23] leveraged an optical shaping technique to realize low bandwidth, sub-Nyquist analog-to-digital conversion in DDM OFDM passive optical networks (PONs). Ref. [24] proposed a novel frequency diversity coded OFDM scheme, which allowed the receiver to sample the received signal at a low rate that was less than the Nyquist rate but also guaranteed a significant diversity–coding gain. Under this condition, an aliasing phenomenon occurred but was regarded as transmission diversity to the receiver. The design criteria of this coding scheme was to minimize the pairwise error probability and the construction frequency diversity code was based on linear block codes. Ref. [25] proposed a simple low sampling rate receiving scheme for a W-band (75–110 GHz) OFDM radio over fiber (RoF) system, which utilized multiple low-sampling-rate ADCs to replace a high-sampling-rate ADC in order to reduce the required sampling rate and the computation complexity of the OFDM demodulation. As for the aliases of the OFDM subcarriers, a digital preprocess that was related to time delays was employed to the low-sampling-rate ADCs to enable all of the subcarriers to be free from aliasing. Ref. [26] discussed a time interleaved (TI) architecture with slower sub-ADCs in parallel, but the mismatch among the sub-ADCs could give rise to error floors in the receiver performance. The authors also proposed an online iterative approach for the joint estimation of mismatch and channel parameters, which leveraged training sequences that were provided for the channel estimation. Ref. [27] presented a DFT/IDFT-free receiving scheme for spread-OFDM signals, which allowed each user to receive the signal without DFT and IDFT using sub-Nyquist sampling and a proper sampling delay. Ref. [28] investigated fronthaul optical links using sub-Nyquist sampling rate ADCs for beyond fifth generation (B5G) and 6G sub-THz massive multiple-input multiple-output (Ma-MIMO) beamforming. Researchers also investigated other possible low sampling rate schemes in different scenarios, such as under-sampling restoration digital predistortion (USR-DPD) [29], frequency comb OFDM radar systems [7] and sub-Nyquist sampling for bandwidth- and hardware-efficient mobile fronthaul [30].

In an OFDM system with *N* subcarriers spaced by 1/T, the dimension of the signal space of one OFDM signal is *N* (or 2N in real dimension) and thus, the Nyquist sampling rate is N/T. When *N* subcarriers are uniformly allocated among *K* users, then the space is divided into *K* subspaces, each with the dimension of V=N/K. In other words, in multi-user OFDM systems, the information intended for each user is roughly constrained within a much smaller subspace. Theoretically, such sparsity implies that fewer measurements (samples) would be needed to retrieve the information, at least for a high SNR regime. Based on this principle, this paper proposes a low sampling rate receiver for multi-antenna multi-user OFDM systems. With the aid of zero-forcing precoding, the sampling rate of each receiver can be reduced to $f_{s}=f_{0}/K$. The simulation results show that the performance loss is insignificant and acceptable in terms of bit error rate, mutual information and peak-to-average power ratio.

The rest of this paper is organized as follows. Section 2 introduces the conventional multi-antenna multi-user OFDM system. Based on this system, we propose a low sampling rate receiver design in Section 3. Section 4 presents the simulation results and comparisons of bit error rate, mutual information and peak-to-average power ratio. Section 5 concludes the paper.

*Notation*: Throughout this paper, we use bold lower case letters to denote vectors, such as a, and bold upper case letters to denote matrices, such as A. $A^{†}$ is the conjugate transpose of matrix A. $A^{*}$ is the element wise conjugate. ∥a∥ is the Euclidean norm. $\mathrm{vect}(a_{1},a_{2},\cdots ,a_{n})$ is the concatenation of *n* column vectors. tr(A) is the trace of A. diag(a) is a diagonal matrix constructed from a. E[·] represents mathematical expectation.

## 2. OFDM Systems

The new design presented in this paper is an upgrade of existing OFDM systems. We first introduce a system model of the existing system in this section. Then, in the next section, we elaborate on the details of our new design.

**Figure 1:**
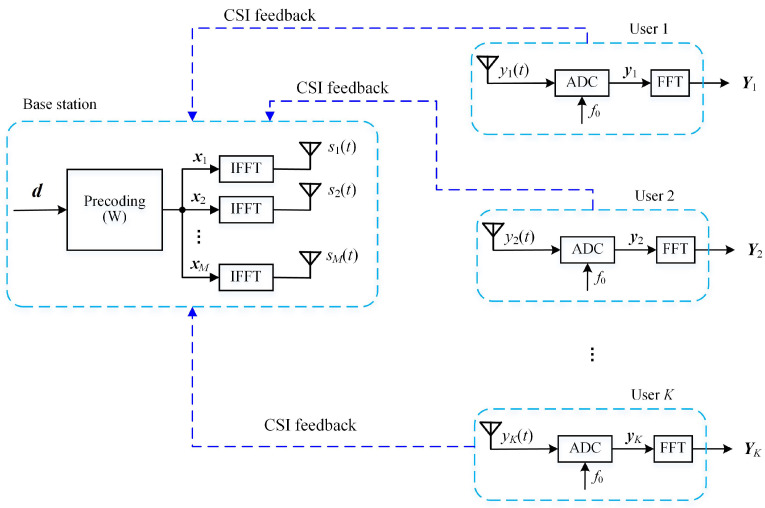
A multi-user OFDM system.

Consider the downlink multi-user OFDM system shown in Figure 1. One base station (BS) is equipped with *M* antennas and *K* users are each equipped with a single antenna. All users share *N* subcarriers of the OFDM system. For simplicity, we assume that *N* is an integer multiple of *K* and that the share of each user is V=N/K.

### 2.1. Signal Model

The data of all users are multiplexed into an aggregate data vector $d={\left(d_{0},d_{1},\cdots ,d_{N-1}\right)}^{T}$, where $d_{n}\in \Omega$ is the data symbol to be transmitted over the *n*-th subcarrier and Ω is the signal constellation, such as QPSK, 16QAM, etc.

To transmit d over *M* transmitting antennas, a precoder W is applied that transforms d into multiple vectors $x_{1},x_{2},\cdots ,x_{M}$, where:

(1) $$x_{m}={\left(x_{m,0},x_{m,1},\cdots ,x_{m,N-1}\right)}^{T}\in C^{N},$$

for m=1,2,⋯,M. The precoded signal is then transmitted over *M* transmitting antennas with $x_{m,n}$ being transmitted over the *n*-th subcarrier and the *m*-th antenna. The time domain signal transmitted at the *m*-th transmitting antenna can be expressed as:

(2) $$s_{m}\left(t\right)=\frac{1}{\sqrt{N}}\sum_{n=0}^{N-1}x_{m,n}e^{j2\pi \frac{n}{T}t},$$

for m=1,2,⋯,M, $-T_{\mathrm{cp}}\leq t\leq T$, where $T_{\mathrm{cp}}$ is the duration of cyclic prefix (CP), *T* is the OFDM symbol period excluding the CP and the orthogonal subcarriers are spaced by 1/T.

The time domain signal received at the *k*-th user is obtained by:

(3) $$\begin{array}{cc} y_{k}\left(t\right) & =\frac{1}{\sqrt{N}}\sum_{m=1}^{M}\sum_{n=0}^{N-1}H_{k,m}^{n}x_{m,n}e^{j2\pi \frac{n}{T}\left(t-\tau_{k}\right)}+z_{k}\left(t\right) \end{array}$$

(4) $$\begin{array}{cc} \; & =\frac{1}{\sqrt{N}}\sum_{m=1}^{M}\sum_{n=0}^{N-1}{\tilde{H}}_{k,m}^{n}x_{m,n}e^{j2\pi \frac{n}{T}t}+z_{k}\left(t\right), \end{array}$$

for k=1,2,⋯,K, where $z_{k}\left(t\right)$ is the additive white Gaussian noise (AWGN), $\tau_{k}$ represents the propagation delay and timing synchronous error and ${\tilde{H}}_{k,m}^{n}=H_{k,m}^{n}e^{-j2\pi \frac{n}{T}\tau_{k}}$ is the overall channel gain. Similar to [31], we assume that the channel state information (CSI), i.e., $\left\{{\tilde{H}}_{k,m}^{n},\forall k,n,m\right\}$, can be estimated by each user and that the estimates can be reported to BS reliably via the signalling channel so that all ${\tilde{H}}_{k,m}^{n},\forall n,k,m$ are known to BS. We will also discuss the effect of imperfect CSI at the BS in Section 4.5.

The ADC shown in Figure 1 converts the continuous waveform into a quantized time-discrete sequence. This paper focuses on the sampling rate and thus, we assume that the quantization error is negligible. Recall that the synchronous error was considered in the overall channel gain ${\tilde{H}}_{k,m}^{n}$ through $e^{-j2\pi \frac{n}{T}\tau_{k}}$; hence, the sampling epochs for each OFDM symbol can be unified as 0,Δ,2Δ,⋯,(N−1)Δ for all users, where Δ=T/N is the sampling interval. Then, the output samples of the ADC with a Nyquist sampling rate $f_{0}=N/T$ can be written as:

(5) $$y_{k}\left(v\right)=\frac{1}{\sqrt{N}}\sum_{m=1}^{M}\sum_{n=0}^{N-1}{\tilde{H}}_{k,m}^{n}x_{m,n}e^{j2\pi \frac{n}{N}v}+z_{k}\left(v\right),$$

for v=0,1,⋯,N−1, where $z_{k}\left(v\right)∼CN\left(0,\sigma^{2}\right)$.

Equation (5) can be expressed in compact matrix form as:

(6) $$y_{k}=F^{†}\left(\sum_{m=1}^{M}\mathrm{diag}({\tilde{H}}_{k,m})x_{m}\right)+z_{k},$$

where $F\in C^{N\times N}$ is the DFT transform matrix:

(7) $$F=\frac{1}{\sqrt{N}}\left(\begin{array}{cccc} 1 & 1 & \cdots & 1\\ 1 & e^{-j\frac{2\pi }{N}} & \cdots & e^{-j\frac{2\pi (N-1)}{N}}\\ \vdots & \vdots & \ddots & \vdots \\ 1 & e^{-j\frac{2\pi (N-1)}{N}} & \cdots & e^{-j\frac{2\pi {\left(N-1\right)}^{2}}{N}} \end{array}\right),$$

${\tilde{H}}_{k,m}\in C^{N}$ is the channel coefficient vector:

(8) $${\tilde{H}}_{k,m}={\left({\tilde{H}}_{k,m}^{0},{\tilde{H}}_{k,m}^{1},\cdots ,{\tilde{H}}_{k,m}^{N-1}\right)}^{T},$$

and $z_{k}\in C^{N}$ is the noise vector.

The ADC output $y_{k}$ is then transformed by FFT, which yields:

(9) $$Y_{k}=Fy_{k}=\sum_{m=1}^{M}\mathrm{diag}\left({\tilde{H}}_{k,m}\right)x_{m}+Z_{k},$$

where $Z_{k}=Fz_{k}$.

### 2.2. Subcarrier Allocation

The total number of *N* subcarriers is uniformly allocated among *K* users and thus, the share of each user is V=N/K. With the knowledge of $\left\{{\tilde{H}}_{k,m}^{n},\forall n,m,k\right\}$, BS can allocate subcarriers to match the user channel condition. In this paper, we assume that the subcarrier *n* is always allocated to the user *k*, which has the largest $\sum_{m=1}^{M}{\left|{\tilde{H}}_{k,m}^{n}\right|}^{2}$ unless the quota of this user is full. In this case, the subcarrier is allocated among remaining users.

### 2.3. Precoding

In the conventional multi-antenna OFDM systems, the precoding is generally performed on a per subcarrier basis, where $x_{m,n}$ is merely a scaling of $d_{n}$:

(10) $$x_{m,n}=d_{n}w_{m,n},$$

for 1≤m≤M,0≤n≤N−1. In case the *n*-th subcarrier is allocated to user *k*, the signal received by user *k* at this subcarrer is the *n*-th element of $Y_{k}$, which can be expressed as:

(11) $$Y_{k,n}=\sum_{m=1}^{M}{\tilde{H}}_{k,m}^{n}x_{m,n}+Z_{k,n}=d_{n}\sum_{m=1}^{M}{\tilde{H}}_{k,m}^{n}w_{m,n}+Z_{k,n},$$

for n=0,1,⋯,N−1,k=1,2,⋯,K. To maximize SNR, the precoding coefficients are set as:

(12) $$w_{m,n}=\frac{{\left({\tilde{H}}_{k,m}^{n}\right)}^{*}}{∥{\tilde{H}}_{k}^{n}∥},$$

for n=0,1,⋯,N−1,m=1,2,⋯,M, where ${\tilde{H}}_{k}^{n}={\left({\tilde{H}}_{k,1}^{n},{\tilde{H}}_{k,2}^{n},\cdots ,{\tilde{H}}_{k,M}^{n}\right)}^{T}$. In other words, the maximal ratio transmission (MRT) [32] is adopted at each subcarrer.

### 2.4. Equivalent Channel Model

Substituting (12) into (11), we obtain the equivalent channel that data $d_{n}$ employ to traverse from BS to user *k* as:

(13) $$Y_{k,n}=∥{\tilde{H}}_{k}^{n}∥d_{n}+Z_{k,n}.$$

The performance of the system can be determined using this model. For example, when $d_{n}$ is drawn from the QPSK constellation, the average BER is then obtained by [33]:

(14) $${\mathrm{BER}}^{\mathrm{OFDM}}=E\left[\frac{1}{2}\mathrm{erfc}\left(\sqrt{∥{\tilde{H}}_{k}^{n}{∥}^{2}\frac{E_{b}}{N_{0}}}\right)\right],$$

and the average mutual information is obtained by [34]:

(15) $$\begin{array}{cc} {\mathrm{MI}}^{\mathrm{OFDM}} & =\;2-\frac{1}{\ln2}-\frac{1}{4}\sum_{i=1}^{4}E\left[\log_{2}\left(\sum_{j=1}^{4}e^{-\frac{{∥Z_{k,n}+∥{\tilde{H}}_{k}^{n}∥\left(s_{i}-s_{j}\right)∥}^{2}}{\sigma^{2}}}\right)\right] \end{array}$$

(16) $$\begin{array}{cc} & \;=\;2-\frac{1}{\ln2}-E\left[\log_{2}\left(\sum_{j=1}^{4}e^{-\frac{{∥Z_{k,n}+∥{\tilde{H}}_{k}^{n}∥\left(s_{1}-s_{j}\right)∥}^{2}}{\sigma^{2}}}\right)\right], \end{array}$$

in bits/symbol, where $s_{i},s_{j}\in \Omega$ are QPSK symbols. The second equality (16) is due to the symmetric property of the QPSK constellation.

## 3. Proposed Low Sampling Rate Design

In the conventional system shown in last section, all users have to use an ADC with a sampling rate that is at least equal to the Nyquist rate. However, the actual frequency bandwidth occupied by each user is only a fraction of the total system bandwidth. This observation inspired the design shown in this section. With the aid of precoding, we significantly reduced the sampling rate of the receiver using this model.

**Figure 2:**
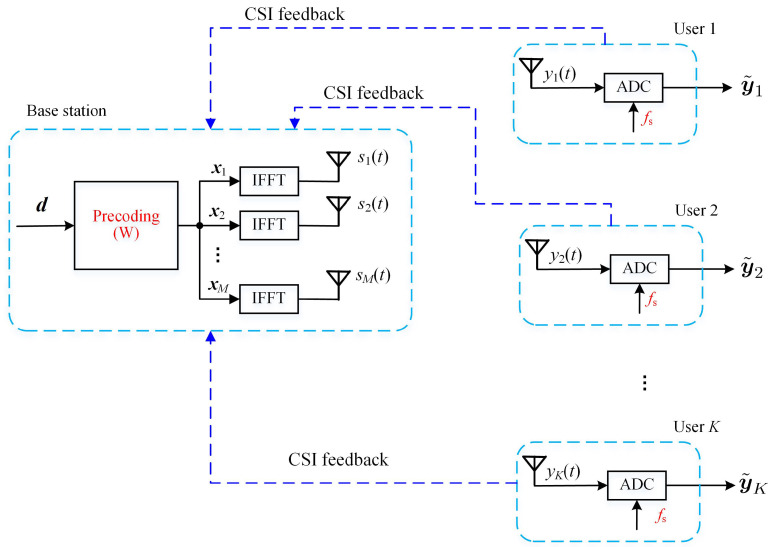
The proposed scheme with a low sampling rate.

The system of the proposed design is shown in Figure 2 and was essentially similar to Figure 1, except for the following points:

1. In the proposed scheme, the sampling rate of the ADC of the receiver was reduced to $f_{s}=f_{0}/K=V/T$;
2. An overall ZF precoder was used at the BS, rather than the per subcarrier MRT precoder as in (12), in order to enable the low sampling rate of the receiver;
3. The FFT module was no longer required at the receiver.

### 3.1. Signal Model

In the proposed scheme, the time domain signal transmitted at the *m*-th antenna is still obtained by (2) and the received signal at the *k*-th user is also obtained by (3), except $x_{m,n}$ is generated in a different way.

With the sampling frequency as $f_{s}=V/T$, the ADC at the *k*-th user output V=N/K samples during the period *T*. The samples could be expressed as:

(17) $${\tilde{y}}_{k}\left(v\right)=\frac{1}{\sqrt{N}}\sum_{m=1}^{M}\sum_{n=0}^{N-1}{\tilde{H}}_{k,m}^{n}x_{m,n}e^{j2\pi \frac{n}{V}v}+{\tilde{z}}_{k}\left(v\right),\;v=0,1,\cdots ,V-1,$$

where ${\tilde{z}}_{k}\left(v\right)∼CN\left(0,\sigma^{2}\right)$. The matrix form of (17) is written as:

(18) $${\tilde{y}}_{k}=D\sum_{m=1}^{M}\mathrm{diag}\left({\tilde{H}}_{k,m}\right)x_{m}+{\tilde{z}}_{k},$$

where ${\tilde{y}}_{k}={\left({\tilde{y}}_{k}\left(0\right),{\tilde{y}}_{k}\left(1\right),\cdots ,{\tilde{y}}_{k}\left(V-1\right)\right)}^{T}$ and ${\tilde{z}}_{k}={\left({\tilde{z}}_{k}\left(0\right),{\tilde{z}}_{k}\left(1\right),\cdots ,{\tilde{z}}_{k}\left(V-1\right)\right)}^{T}$. ${\tilde{H}}_{k,m}$ is the same as in (8). Similar to the conventional OFDM system in Figure 1, we also assume that each user could perfectly estimate its channel $\left\{{\tilde{H}}_{k,m}^{n},\forall n,m\right\}$ and feed back the measurements to the BS. Note that the low sampling rate of the receiver does not mean that we encounter difficulties in channel estimation. In fact, due to the sparsity of the channel responses, the channel estimation in real OFDM systems is generally performed on a subset of subcarriers [35].

The matrix $D\in C^{V\times N}$ in (17) is obtained by:

(19) $$D=\frac{1}{\sqrt{N}}\left(\begin{array}{cccc} 1 & 1 & \cdots & 1\\ 1 & e^{j\frac{2\pi }{V}} & \cdots & e^{j\frac{2\pi (N-1)}{V}}\\ \vdots & \vdots & \ddots & \vdots \\ 1 & e^{j\frac{2\pi (V-1)}{V}} & \cdots & e^{j\frac{2\pi (V-1)(N-1)}{V}} \end{array}\right).$$

Note that D is known beforehand by the BS.

### 3.2. Precoding

Since D is rank deficient, we could not perform a transform $D^{†}$ on ${\tilde{y}}_{k}$ to recover data, as we could in (9). Thus, we resort to precoding.

We define:

(20) $$C_{k,m}=D\mathrm{diag}\left\{{\tilde{H}}_{k,m}\right\},$$

for k=1,2,⋯,K and m=1,2,⋯,M. Then, (18) could be rewritten as:

(21) $${\tilde{y}}_{k}=\sum_{m=1}^{M}C_{k,m}x_{m}+{\tilde{z}}_{k},$$

for k=1,2,⋯,K. We further define:

(22) $$\begin{array}{cc} \;x= & \mathrm{vect}\left(x_{1},x_{2},\cdots ,x_{M}\right)\in C^{MN}, \end{array}$$

(23) $$\begin{array}{cc} \tilde{y}= & \mathrm{vect}\left({\tilde{y}}_{1},{\tilde{y}}_{2},\cdots ,{\tilde{y}}_{K}\right)\in C^{N}, \end{array}$$

(24) $$\begin{array}{cc} \tilde{z}= & \mathrm{vect}\left({\tilde{z}}_{1},{\tilde{z}}_{2},\cdots ,{\tilde{z}}_{K}\right)\in C^{N}, \end{array}$$

(25) $$\begin{array}{cc} \;C= & \left(\begin{array}{cccc} C_{1,1} & C_{1,2} & \cdots & C_{1,M}\\ C_{2,1} & C_{2,2} & \cdots & C_{2,M}\\ \vdots & \vdots & \ddots & \vdots \\ C_{K,1} & C_{K,2} & \cdots & C_{K,M} \end{array}\right)\in C^{N\times MN}. \end{array}$$

Then, the signals received by all users could be compactly written as:

(26) $$\begin{array}{cc} \tilde{y}= & Cx+\tilde{z} \end{array}$$

(27) $$\begin{array}{cc} \;= & CWd+\tilde{z}, \end{array}$$

where $W\in C^{MN\times N}$ is the precoding matrix. $d=\mathrm{vect}(d_{1},d_{2},\cdots ,d_{k})$, in which $d_{k}\in \Omega^{V}$ is the data symbol intended for user *k*.

Equation (27) indicates that the whole system in Figure 2 could be regarded as an virtual MIMO system with C as its channel gain matrix. Through the appropriate design of W, the interference among users could be canceled out at each receiver.

For simplicity, the transmit zero-forcing [36] precoding is considered here. The ZF precoder is obtained by:

(28) $$W_{\mathrm{ZF}}=cC^{†}{\left(CC^{†}\right)}^{-1},$$

where *c* is the normalization factor:

(29) $$c=\sqrt{\frac{N}{\mathrm{tr}\left(C^{†}{\left(CC^{†}\right)}^{-1}{\left(C^{†}{\left(CC^{†}\right)}^{-1}\right)}^{†}\right)}},$$

so that $E[∥W_{\mathrm{ZF}}{\;x∥}^{2}{]\;=E[∥x∥}^{2}]$.

### 3.3. Equivalent Channel Model

Substituting (28) into (27), we obtain:

(30) $$\tilde{y}=cd+\tilde{z}.$$

For the *k*-th user, the *v*-th sample is:

(31) $${\tilde{y}}_{k}\left(v\right)=cd_{k}\left(v\right)+{\tilde{z}}_{k}\left(v\right),$$

for v=0,1,⋯,V−1 and k=1,2,⋯,K. This means that the outputs of the ADC are directly the desired data symbols, with scaling and polluted by AWGN. This is the reason that we have omitted the FFT module from Figure 2.

Similar to (14) and (16), the average BER and average MI of the proposed system are obtained by:

(32) $${\mathrm{BER}}^{\mathrm{Prop}}=E\left[\frac{1}{2}\mathrm{erfc}\left(\sqrt{c^{2}\frac{E_{b}}{N_{0}}}\right)\right]$$

and

(33) $${\mathrm{MI}}^{\mathrm{Prop}}=2-\frac{1}{\ln2}-E\left[\log_{2}\left(\sum_{j=1}^{4}e^{-\frac{{∥{\tilde{z}}_{k}\left(v\right)+c\left(s_{1}-s_{j}\right)∥}^{2}}{\sigma^{2}}}\right)\right],$$

respectively.

## 4. Simulation Results

In the last section, we proposed a new design that could significantly reduce the sampling rate of the receiver. We did not expect this advantage to come for free, but we hoped that the performance loss would be acceptable. In this section, we use simulations to verify this.

### 4.1. Simulation Setting

In the simulation, the data symbols were drawn from the QPSK constellation. The number of transmitting antennas was M=2 or 4. The number of users was K=2, 4, 8 or 16. The number of subcarriers ranged from N=64 to 256.

An 8-tap time-delay-line channel model was used for the channel from the *m*-th transmitting antenna to the *k*-th user. The time domain channel response was:

(34) $$h_{k,m}={\left[h_{k,m}^{0},h_{k,m}^{1},\cdots ,h_{k,m}^{7}\right]}^{T},$$

for all k=1,2,⋯,K and m=1,2,⋯,M. All $\left\{h_{k,m}^{l}\right\}$ were independently drawn from the complex Gaussian distribution CN(0,1) and then each $h_{k,m}$ was normalized, such that $∥h_{k,m}{∥}^{2}=1$. The frequency domain channel coefficients were obtained by performing FFT on $h_{k,m}$, i.e.,

(35) $$H_{k,m}=\sqrt{N}F\;\mathrm{vect}\left(h_{k,m},0_{N-8}\right),$$

where $0_{N-8}$ is an all zero vector of length N−8.

The synchronization error (including propagation delay) $\tau_{k}$ for the *k*-th user was uniformly distributed in the sampling interval. Specifically, $\tau_{k}$ was uniformly distributed in $\left[-\frac{T}{2N},\frac{T}{2N}\right]$ for the OFDM system in which the sampling interval was T/N and it was uniformly distributed in $\left[-\frac{T}{2V},\frac{T}{2V}\right]$ for the proposed system in which the sampling interval was T/V.

### 4.2. BER

**Figure 3:**
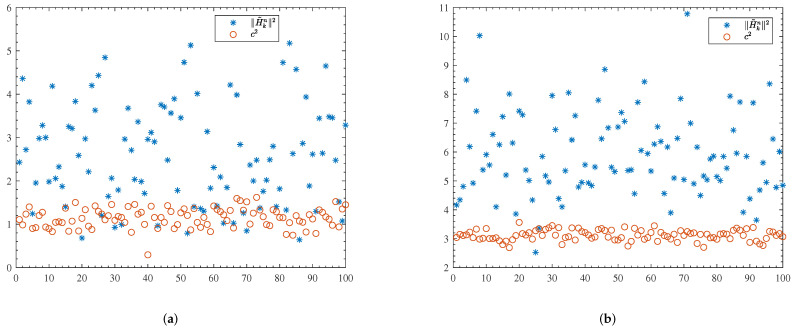
Random samples of $∥{\tilde{H}}_{k}^{n}{∥}^{2}$ and $c^{2}$. (**a**) N=64, M=2, K=2; (**b**) N=64, M=4, K=4.

We can observe form (14) and (32) that the difference in BER performance lies in the difference between $∥{\tilde{H}}_{k}^{n}{∥}^{2}$ and $c^{2}$. For the conventional system, both subcarrier selection and MRT precoding can bring the diversity gain and array gain [33]. For the proposed scheme, *c* is the scaling coefficient of the ZF precoder. We know that when ZF is used as receiver equalizer, it has the drawback of noise enhancement. This drawback turns out to be the transmit power penalty when it is used as a transmit precoder. However, *c* is involved with all channel coefficients on all subcarriers and therefore, we could expect a larger diversity gain. In Figure 3, we present 100 random realizations of $∥{\tilde{H}}_{k}^{n}{∥}^{2}$ (blue asterisks) and $c^{2}$ (brown circles). $∥{\tilde{H}}_{k}^{n}{∥}^{2}$ was in general larger than $c^{2}$, indicating a larger array gain; while $c^{2}$ was less fluctuating, indicating a larger diversity gain.

**Figure 4:**
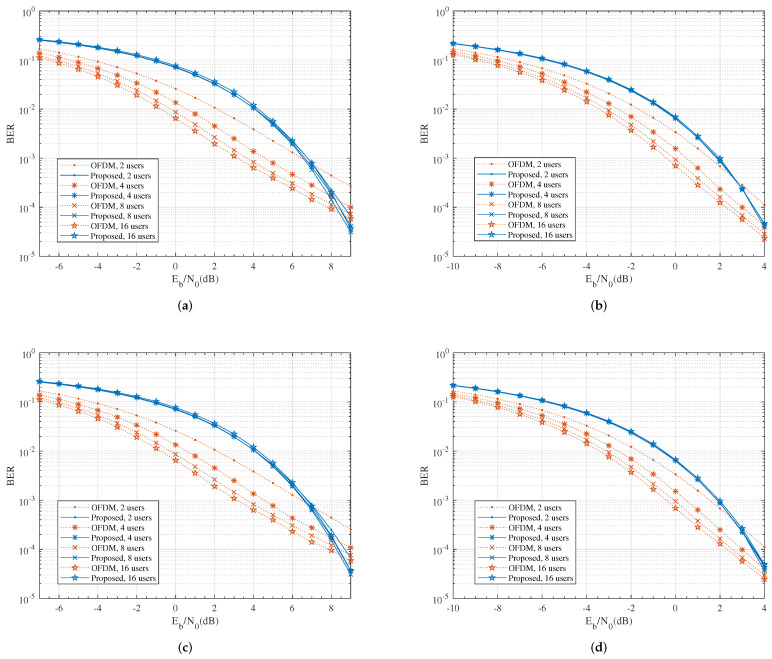
The comparison of BER performance for uncoded QPSK modulation. (**a**) M=2, N=64; (**b**) M=4, N=64; (**c**) M=2, N=256; (**d**) M=4, N=256.

Figure 4 compares the simulated BER performance of the proposed system to the OFDM system. As expected, in the high SNR regime, the BER of the proposed scheme decayed much faster than the conventional OFDM system due to the larger diversity gain; while in the low to medium SNR regime, OFDM had better performance due to the array gain. At BER = $10^{-4}$, both systems produced a similar performance.

**Figure 5:**
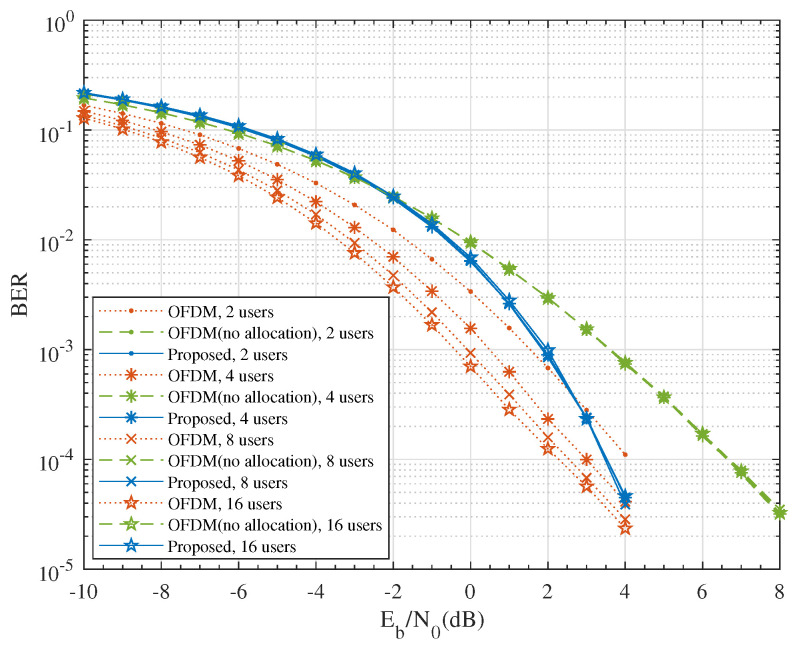
The effect of subcarrier allocation on BER performance for uncoded QPSK modulation with M=4 and N=64.

The array gain of the OFDM system comes from the subcarrier selection diversity, which is shown in Figure 5. The performance of the OFDM system without subcarrier allocation was worse than that of the proposed scheme.

### 4.3. MI

**Figure 6:**
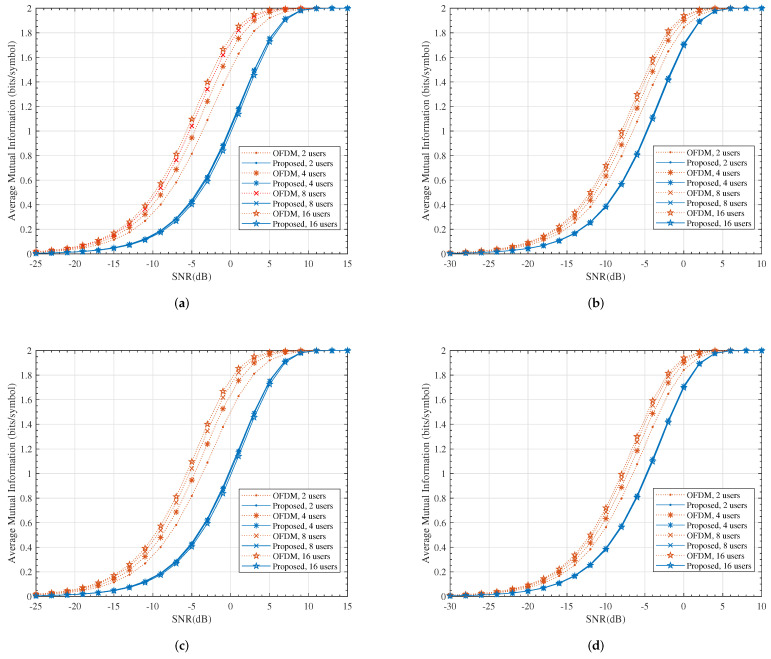
The comparison of average mutual information. (**a**) M=2, N=64; (**b**) M=4, N=64; (**c**) M=2, N=256; (**d**) M=4, N=256.

Figure 6 compares the numerical results of (16) and (33). The average mutual information represents the maximum data rate, which could be achieved using infinitely long ergodic ideal code (capacity achieving code). Channel code has an inherent diversity capability with a diversity order that is equal to the minimum code distance and thus, with the ideal channel code, the array gain dominates the performance. This can be observed in Figure 6. Due to the loss in array gain, our proposed scheme had poor MI performance compared to the OFDM system and the corresponding SNR loss ranged from 2∼5 dB. This loss was mainly on account of the power penalty of the ZF precoder and hence, it could be remedied by using alternative precoders, such as DPC, BD-SLNR or MMSE-SLNR [37,38].

**Figure 7:**
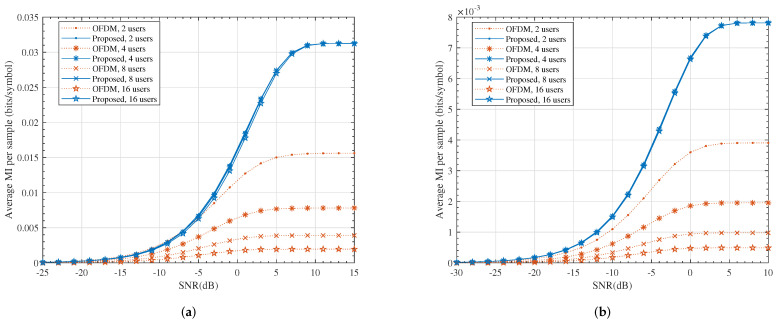
The average mutual information per sample. (**a**) M=2, N=64; (**b**) M=4, N=256.

It is worth noting that *K* users had NK samples altogether at the output of the ADC units in the conventional OFDM system, while this number was *N* for the proposed system. In other words, the samples in the proposed system were much more informative. This can be observed in Figure 7.

### 4.4. PAPR

**Figure 8:**
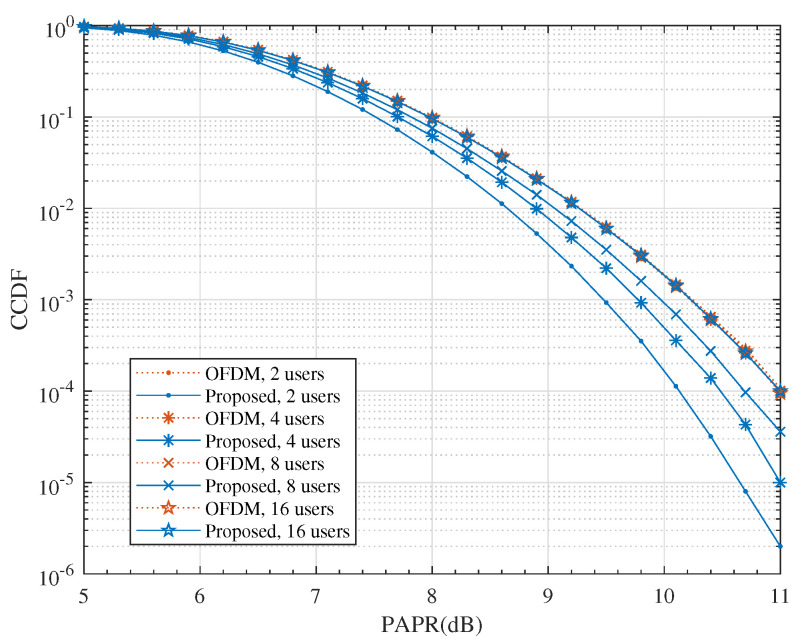
The comparison of CCDF for QPSK modulation with M=2 and N=64.

Another concern was whether the proposed scheme would raise the PAPR of the transmit signal. High PAPR increases the hardware costs of power amplifiers (PAs) and ADCs since it has to increase the resolution to cope with the high dynamic input signal. The simulated CCDF of PAPR for both systems are shown in Figure 8. We can see that the proposed scheme did not raise the PAPR of the transmission signal and it even produced some gain over the OFDM system.

### 4.5. Imperfect CSI

In this subsection, we consider the effect of imperfect CSI at the BS. Specifically, we assumed that perfect CSI, i.e., $\left\{{\tilde{H}}_{k,m}^{n},\forall k,n,m\right\}$, was available for each user through channel estimation and reporting to the BS via the signalling channel. However, the CSI obtained by the BS might not be accurate due to the limited feedback. For simplicity, the imperfect CSI obtained by the BS was formulated as:

(36) $${\hat{H}}_{k,m}^{n}={\tilde{H}}_{k,m}^{n}+\varepsilon ,$$

for k=1,2,⋯,K, n=1,2,⋯,N and m=1,2,⋯,M, where ε is the CSI error, ε∼CN(0,MSE) and MSE is the mean square error of the CSI error, $MSE=E\left[|{\hat{H}}_{k,m}^{n}-{\tilde{H}}_{k,m}^{n}{|}^{2}\right]$. Under this condition, the OFDM system and the proposed scheme used the imperfect CSI to perform the precoding instead, i.e., $\left\{{\hat{H}}_{k,m}^{n},\forall k,n,m\right\}$.

**Figure 9:**
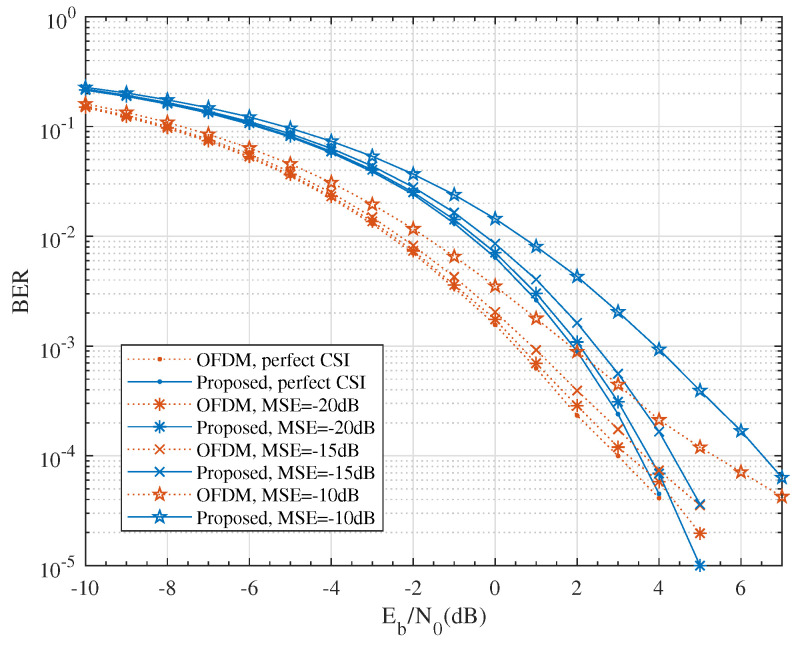
The effect of imperfect CSI on BER performance for QPSK modulation with M=4, N=64 and K=4.

As the numerical results show in Figure 9, the effect of imperfect CSI was subtle when the MSE was small, for example, MSE=−20dB. Specifically, compared to perfect CSI at the BS, the BER performance loss with MSE=−20dB, −15dB and −10dB at $\mathrm{BER}=10^{-4}$ were 0.19 dB, 0.58 dB and 2.39 dB for the OFDM system and 0.27 dB, 0.83 dB and 3.06 dB for the proposed scheme, respectively. Both schemes produced a similar performance loss and were robust at a moderate level of CSI error.

## 5. Conclusions

This paper proposed a low sampling rate receiver design using zero-forcing precoding for multi-antenna multi-user OFDM systems. Compared to the traditional OFDM system, the proposed scheme achieved a low sampling rate at the receiver and no FFT operation was required on the receiving side. The proposed scheme significantly reduced the complexity of the receivers and thus, would contribute to the design of low cost terminals. The simulation results have shown that the proposed scheme produced an acceptable performance in terms of bit error rate, mutual information and peak-to-average power ratio.
